# Supplementary material for: Role of the Photorhabdus Dam methyltransferase during interactions with its invertebrate hosts
Source: PLoS One. 2019 Oct 9;14(10):e0212655. doi: 10.1371/journal.pone.0212655 (PMC6785176; doi:10.1371/journal.pone.0212655)
Supplement: S1 Fig — (PDF) [file pone.0212655.s001.pdf]

Fig. S1

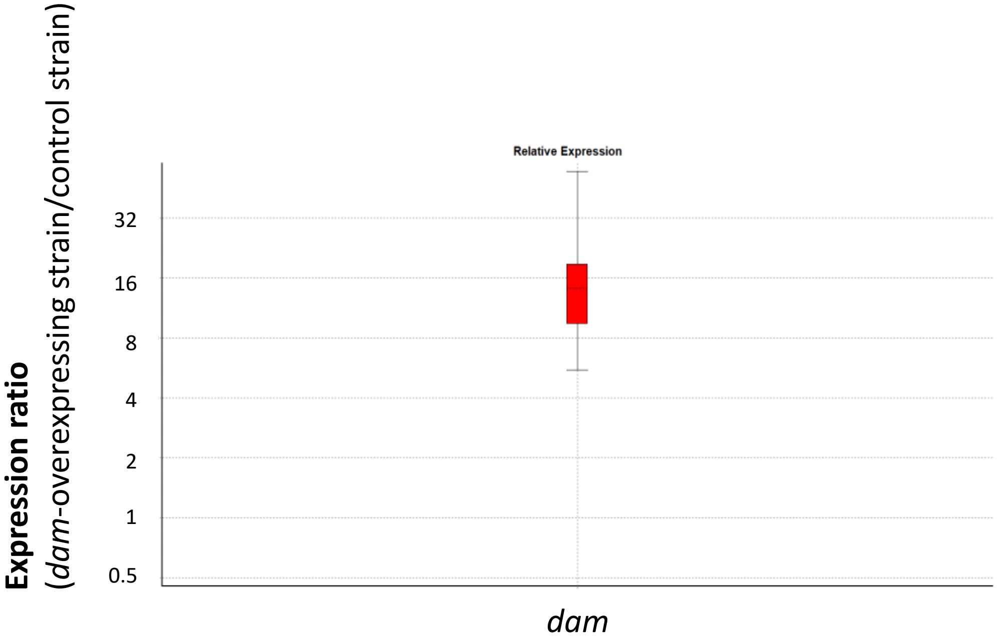

Relative Expression Results

Parameter	Value
Iterations	2000

Gene	Type	Reaction Efficiency	Expression	Std. Error	95% C.I.	P(H1)	Result
GyrB	REF	0,7929	1,157				
Dam	TRG	1,1132	14,217	8,224 - 23,010	5,785 - 41,381	0,000	UP
fis	REF	0,7791	0,864				

Interpretation

Dam is UP-regulated in sample group (in comparison to control group) by a mean factor of 14,217 (S.E. range is 8,224 - 23,010).

Dam sample group is different to control group. P(H1)=0,000

Figure S1. Relative expression of the *dam* gene in the *P. luminescens dam*-overexpressing strain.
qRT-PCR was carried out with total RNA extracted from 3 independent samples of exponentially growing cells of the *dam*-overexpressing strain and of the control strain. Left panel, Box-plot representing the expression ratio between the two strains is represented for the *dam* gene. Right panel, detailed report of the REST-analysis using with *gyrB* and *fis* used as control genes (see Materials & Methods section for details). The level of expression between the 2 strains was different at p<0.001.
