# Supplementary material for: Role of the Photorhabdus Dam methyltransferase during interactions with its invertebrate hosts
Source: PLoS One. 2019 Oct 9;14(10):e0212655. doi: 10.1371/journal.pone.0212655 (PMC6785176; doi:10.1371/journal.pone.0212655)
Supplement: S2 Fig — (PDF) [file pone.0212655.s002.pdf]

Fig. S2

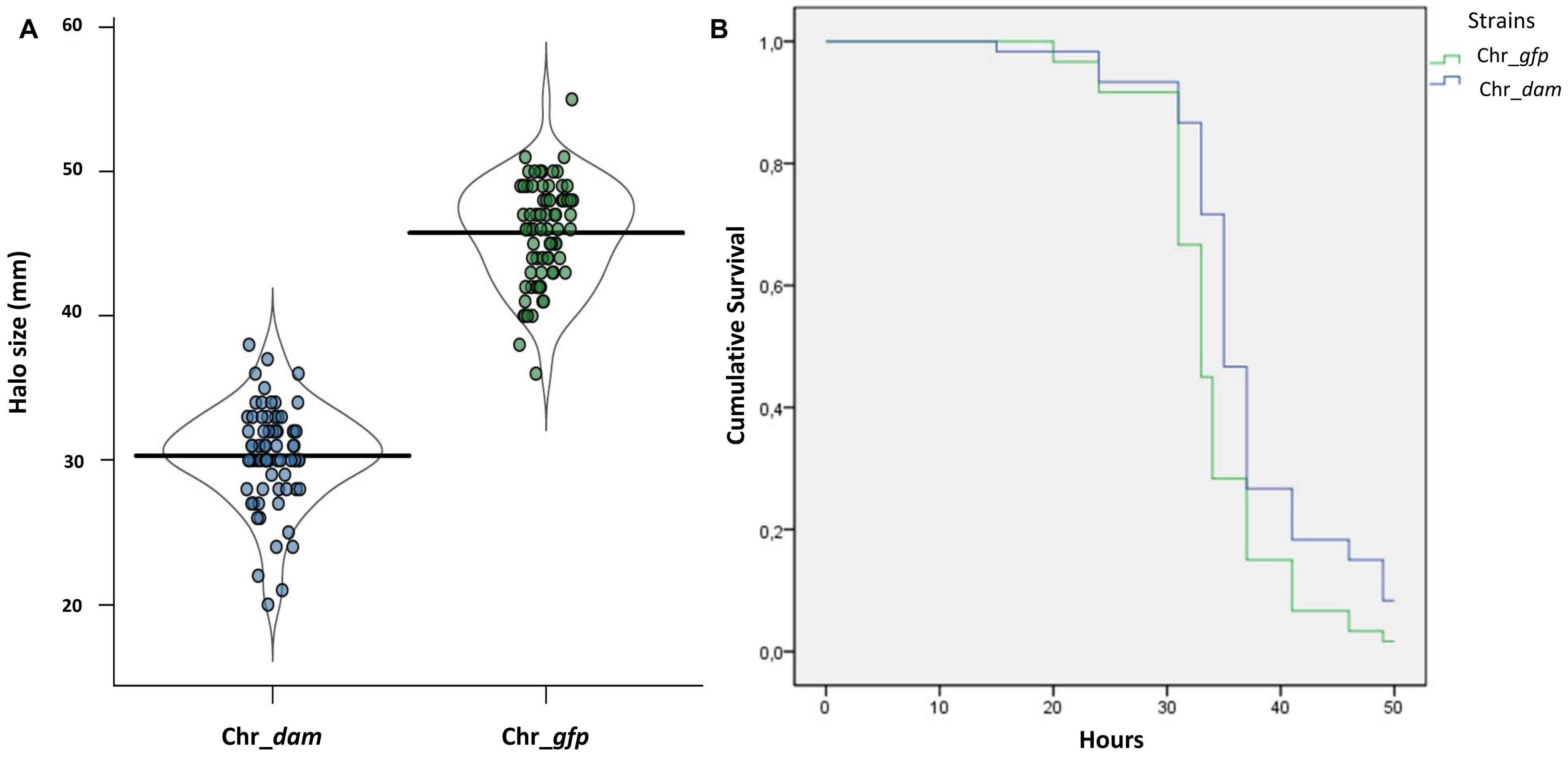

Figure S2. Motility and pathogenicity of *Chr_dam* strain.
(A) Violin-plot of motility halo size for Chr_*dam* and Chr_*gfp* strain after 36 hours of growth on motility medium. The difference between the two strains was significant (Wilcoxon test, p-value<0.001). (B) Survival of *S. littoralis* larvae after injection of 10^4 CFU of Chr_*gfp* (green) or Chr_*dam* (blue). Chr_*dam* strain was significantly delayed (2hours) in the time needed to kill 50 % of the larvae (Wilcoxon test, p-value<0.001).
