# Supplementary material for: Role of the Photorhabdus Dam methyltransferase during interactions with its invertebrate hosts
Source: PLoS One. 2019 Oct 9;14(10):e0212655. doi: 10.1371/journal.pone.0212655 (PMC6785176; doi:10.1371/journal.pone.0212655)
Supplement: S3 Fig — (PDF) [file pone.0212655.s003.pdf]

Fig. S3

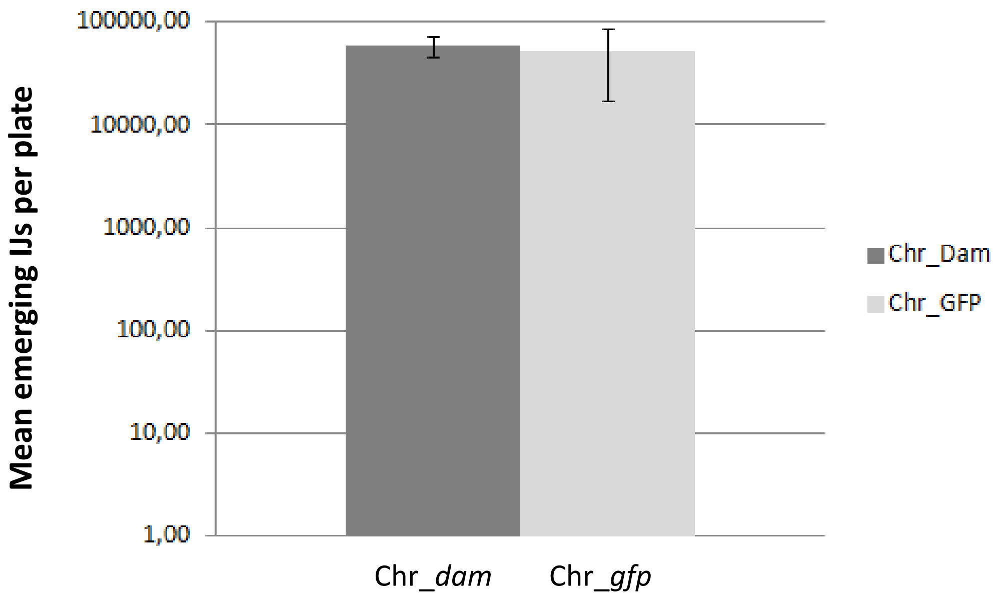

Figure S3. Emerging IJs from in vitro symbiosis association.
For each *P. luminescens* strain, three independent cultures were used to establish symbiosis with *H. bacteriophora*. Emerging IJs were then counted and the mean of three biological replicates is represented for Chr_*dam* strain (dark grey) and Chr_*gfp* (light grey) (see Materials & Methods section for details). The level of emergence between the 2 strains was not significantly different (Wilcoxon, p=0.63).
