# Supplementary material for: Role of the Photorhabdus Dam methyltransferase during interactions with its invertebrate hosts
Source: PLoS One. 2019 Oct 9;14(10):e0212655. doi: 10.1371/journal.pone.0212655 (PMC6785176; doi:10.1371/journal.pone.0212655)
Supplement: S1 Table — (PDF) [file pone.0212655.s004.pdf]

Table S1: Primers used in this study

Primer name	Use	Sequence (5'- 3')
R_GlmS_SalI	Partial amplification of *GlmS* for chromosomal insertion	GCGGTCGACTTCAACTAAGGCATTTACAAC
F_GlmS_AatII	Partial amplification of *GlmS* for chromosomal insertion	GGCGACGTCAACCTTATTCCACCGTCAC
R_RpmE_SacI	Partial amplification of *RpmE* for chromosomal insertion	GGCGAGCTCTAGTAAGAAGTTGAAATAAGCC
F_RpmE_SpeI	Partial amplification of *RpmE* for chromosomal insertion	GCTACTAGTTAAACCCGCAGTTATAAGC
L_verif_GlmS	PCR verification of chromosomal insertion	TGCGGATACATTAGCAGCAC
R_verif_RpmJ	PCR verification of chromosomal insertion	GCACTAATAAGGTTTCATTTTGCAT
plu0004-F	qRT-PCR on *gyrB* gene	ATACACGAAGAAGAAGGTGTTTCAG
plu0004-R	qRT-PCR on *gyrB* gene	TACCTGTCTGTTCAGTTTCTCCAAC
plu087-F	qRT-PCR on *dam* gene	CTGATGAGTTTATAGTCCACACACG
plu087-R	qRT-PCR on *dam* gene	ACCATGAGAATTATAACGACAGAGG
MSRE-10531-F	Determination of the DNA methylation status	CAGCACTGACTCTGGAATTAAAAAT
MSRE-10531-R	Determination of the DNA methylation status	CCCTTAACTAAAACATGAACTCTCA
