# Supplementary material for: Role of the Photorhabdus Dam methyltransferase during interactions with its invertebrate hosts
Source: PLoS One. 2019 Oct 9;14(10):e0212655. doi: 10.1371/journal.pone.0212655 (PMC6785176; doi:10.1371/journal.pone.0212655)
Supplement: S2 Table — (PDF) [file pone.0212655.s005.pdf]

Table S2: **Phenotypes of *P. luminescens* TT01 Chr_*dam* and Chr_*gfp* strains**

Strain[a]	Btb adsorption[b]	Bioluminescence[c]	Antibiotic production[d]	Sheep blood hemolysis[e]	Motility[f]	lecithinase[g]	Lipolysis of[h]				
							Tween 20	Tween 40	Tween 60	Tween 80	Tween 85
TT01 Chr_Dam	G	+	+	-	+	+	+	+	+	+	-
TT01 Chr_GFP	G	+	+	-	++	+	+	+	+	+	-
TT01 WT	G	+	+	-	++	+	+	+	+	+	-

[a] All plates were incubated for 2 days at 28°C before assays were interpreted, unless otherwise indicated. Routinely tested phenotypes on the WT strain are indicated for comparison.

[b] Btb, bromothymol blue; G, green-blue colonies on NBTA medium.

[c] +, Luminescence detected by visual observation in a dark room.

[d] +, Halo size (>10 mm) of growth inhibition of *Micrococcus luteus*.

[e] -, No halo of hemolysis detected

[f] +, Reduced spreading area (halo size < 40 mm); ++, Large spreading area (halo size > 40 mm) after 36h of incubation.

[g] +, Halo of opacity around the inoculum indicated the production of lecithinase

[h] +, Halo of precipitation; -, no halo of precipitation
